# Research Blogging: Indexing and Registering the Change in Science 2.0

**DOI:** 10.1371/journal.pone.0050109

**Published:** 2012-12-12

**Authors:** Sibele Fausto, Fabio A. Machado, Luiz Fernando J. Bento, Atila Iamarino, Tatiana R. Nahas, David S. Munger

**Affiliations:** 1 Escola de Comunicações e Artes, Universidade de São Paulo, São Paulo, Brazil; 2 Instituto de Biociências, Universidade de São Paulo, São Paulo, Brazil; 3 Instituto de Biologia, Universidade Federal do Rio de Janeiro, Rio de Janeiro, Brazil; 4 Instituto de Ciências Biomédicas, Universidade de São Paulo, São Paulo, Brazil; 5 São Paulo, Brazil; 6 New York, New York, United States of America; University of Maribor, Slovenia

## Abstract

Increasing public interest in science information in a digital and 2.0 science era promotes a dramatically, rapid and deep change in science itself. The emergence and expansion of new technologies and internet-based tools is leading to new means to improve scientific methodology and communication, assessment, promotion and certification. It allows methods of acquisition, manipulation and storage, generating vast quantities of data that can further facilitate the research process. It also improves access to scientific results through information sharing and discussion. Content previously restricted only to specialists is now available to a wider audience. This context requires new management systems to make scientific knowledge more accessible and useable, including new measures to evaluate the reach of scientific information. The new science and research quality measures are strongly related to the new online technologies and services based in social media. Tools such as blogs, social bookmarks and online reference managers, Twitter and others offer alternative, transparent and more comprehensive information about the active interest, usage and reach of scientific publications. Another of these new filters is the Research Blogging platform, which was created in 2007 and now has over 1,230 active blogs, with over 26,960 entries posted about peer-reviewed research on subjects ranging from Anthropology to Zoology. This study takes a closer look at RB, in order to get insights into its contribution to the rapidly changing landscape of scientific communication.

## Introduction

The instruments and methodologies from Bibliometrics and Scientometrics traditionally cooperate in and are widely used by development agencies, academic institutions, and even corporations for planning and management of policies for Science and Technology (S&T), identification and promotion of new areas of research, and many other issues in strengthening and growth of S&T activities.

Bibliometrics and Scientometrics tools provide statistics and indicators to generate measures of published scientific output. Although admittedly imperfect [1]–[3], this field is mainly based on the number of publications and citations. In fact, as S. Arbesman has written,

For too long, the measurement of scientific contribution has centered on the publication. Whether through the number of articles, the citations those articles have by other articles, or even other far more complicated metrics, most scientists are still measured by a derivative of the research article, the basic technology of scientific publishing that is well over 300 years old [4].

This is a more than 300 year-old *modus operandi* of science communication, which began with the invention of the scientific journal in the 17^th^ century [5] and was well suited to communicating scientific research results for a long time in a world where scientists published their findings, theories and ideas to other scientists. But it is insufficient for the current context of an increasing public interest in science information in a digital and 2.0 science era, where the scientific community is witnessing a dramatic, rapid and deep change. The emergence and expansion of information and communication technologies and internet-based tools is opening space for new possibilities to improve both scientific methodology and communication, assessment, promotion and certification [6].

New technologies allow modern methods of acquisition, manipulation and storage, generating massive data volumes that can further facilitate the research process [7],[8]. These technologies also facilitate access to scientific results through information sharing and discussion. Content previously restricted only to specialists is now available to a wider audience.

This context requires new management systems to make scientific knowledge more accessible and useable, including new measures to evaluate the reach of scientific information not only among professionals and specialists but also to the general public. The new science and research quality measures are strongly related to the new online technologies and services based in social media. Tools such as blogs, social bookmarks, online reference managers (CiteULike, Connotea, Mendeley, Zotero), and Twitter offer alternative, transparent and more comprehensive information about the active interest, usage and reach of scientific publications [9]–[15]. External online tools also represent a new form of post-publication review (*e.g.* Wikipedia referencing of articles is an indicator of future citations [16]), a result of the filtering done by specialist authors.

All these changes are stimulating the scientific community to reassess its means of communication. For example, the Science Online conference, now in its sixth edition (in January 2012) aims to explore science on the web [17], encouraging studies have been released [18], alternative metrics as PLoS Article-Level Metrics have been developed [19]–[21], and all of these developments have helped to grow movements such as the new field of Altmetrics [22]. These new tools are based on a belief in the failure and insufficiency of the three more traditional filters - peer-review, citation counting analysis, and Journal Impact Factor - to indicate the most relevant and significant sources in a context of an explosive growth of the volume of academic literature in today's internet-age science.

Here we highlight scientific blogs as one important new filter of scientific research. The science blogosphere has grown significantly in recent years. The information gap that was traditionally fulfilled by science journalists and scientifically-curious laymen now has a new protagonist: the scientist. Blogs are one of the most common methods that scientists use to communicate their ideas to other scientists or to the general public [23]. This preference may be due to incentives for scientists to engage with the blogosphere [24] and face its challenges to traditional peer-reviewed research channels. But these challenges may also be a great opportunity [25], enabling scientists to make a direct connection to students [26]–[28] and bringing them closer to the general public. Scientific blogs have a positive tendency for aggregation, mainly through blog platforms developed by respected science journals or through new tools that either allow a new system of science publishing [29] and post-publication filtering or value online peer-reviewed publication.

This study aims to describe the platform Research Blogging, an aggregator of scientific blog citations of peer-reviewed publications, showing its history, current configuration and characterization of languages, covered topics, number of blogs, posts, use of Open Access (OA), and mentions of scientific and other research. We see it as a critical tool in the ever-changing world of scientific communication, with its own important contribution to this change in the science endeavor.

### Research Blogging: background, current state and characterization

Research Blogging (RB) was created in 2007 by the scientific blogger Dave Munger, after one of his readers showed appreciation for his use of an icon to distinguish posts about peer-reviewed research from other general or personal messages on his blog. An icon for all scientific blog posts citing peer-reviewed research was developed, and then a central aggregator collected all such marked posts in a collection harvested from across the internet. Soon, hundreds of bloggers were using the site and a new platform [http://researchblogging.org] was developed and is still maintained in collaboration with Seed Media Group. The RB Website aggregates peer-reviewed research posts from several science blogs in seven different languages: English, Spanish, Portuguese, German, Chinese, Polish and Italian. It is a useful source for readers interested in cutting-edge research and first-hand comments and explanations of science, by scientists and experts in their respective fields. In addition, given that the intrinsic structure of the web makes it difficult to a clear distinction between scientific and pseudo-scientific content, RB is a tool to identify serious academic research and avoid the spread of pseudo-scientific contents, serving as a self-regulated organization that helps to collect only academically relevant information. The site now has over 1,230 active blogs, with over 26,960 entries posted about peer-reviewed research on subjects ranging from Anthropology to Zoology, in categorized blogs.

### How Research Blogging works

All RB content is user generated. Participating bloggers - often experts in their research area - identify relevant research in their field. When they write substantive posts about the research on their blogs, they can choose to have those posts aggregated by RB. RB serves as a central means of disseminating findings of peer-reviewed research that careful bloggers have found interesting enough to read and closely analyze.

After registration, bloggers decide themselves to which category their blog will belong indicating their blog topics from the available list within RB site:

Anthropology

Astronomy

Biology

Chemistry

Computer Science/Engineering

Ecology/Conservation

Geosciences

Health

Mathematics

Medicine

Neuroscience

Philosophy

Physics

Psychology

Social Science

Research/Scholarship

or Other

Once registered in RB, bloggers use a one-line form to create a snippet of code to place in their posts. This snippet not only notifies the RB site about the scientific posts, it also creates a properly formatted research citation for the blog. The RB software automatically scans registered blogs for posts containing RB code snippet. When it finds them, it indexes and displays them on site front page — thousands of posts from hundreds of blogs, organized by topic. RB editors identify the notable posts in each major discipline, publishing the results on news page in the platform. Other services like PubGet [http://pubget.com] index the RB database as well, so every time readers search for a journal article, they can also locate blog posts discussing the article, and RB also uses sharing tools for divulgation through RSS feeds and social media applications (app) as Twitter.

### Quality Control

Participating bloggers agree to use the “Blogging on Peer-Reviewed Research” icons and the aggregator at ResearchBlogging.org only when they are writing a thoughtful, original blog post about peer-reviewed research. Just a linking to or quoting a news article or press release is not considered sufficient for inclusion on RB.

Blogs can be a powerful tool for dissemination of scientific information and RB is one of the tools that promote a self-regulated quality control of blog posts. Bloggers must demonstrate to the RB editors and readers that they regularly produce posts that meet the criteria to use a “blog badge” [28]. RB editors ensure that newly-registered blogs follow guidelines based on weeks of discussion at ResearchBlogging.org community to safeguard the quality of the aggregator platform. The site continues to receive further recommendations and suggestions for modifications to these guidelines, which are subject to ongoing revision so as to maintain the spirit of good scholarship. The quality of the posts listed on RB site is monitored by the blogger members. If a post doesn't follow the guidelines, it is removed from RB database, and borderline cases may be discussed publicly on the RB blog as well.

The following extract, taken directly from the RB site, describes the most important guidelines for inclusion:

1. The “Blogging on Peer-Reviewed Research” icons are to be used solely to denote individual blog posts about peer-reviewed research;
2. Similarly, when a blogger is registered with ResearchBlogging.org and uses our system to generate a citation for purposes of aggregation by our site, the citation is to be used solely to denote individual blog posts about the peer-reviewed research listed in the citation;
3. While there is no hard-and-fast definition of “peer-review,” peer reviewed research should meet the following guidelines:
  *Reviewed by experts in field
  *Edited
  *Archived
  *Published with clearly stated publication standards
  *Viewed as trustworthy by experts in field
  *In the case of certain curated archives such as arXiv.org, the “intention” for research to be reviewed may be seen as an adequate proxy for peer review
4. Posts using the icon or RB citation code should offer a complete formal citation of the work(s) being discussed;
5. The post author should have read and understood the entire work cited;
6. The blog post should report accurately and thoughtfully on the research it presents;
7. Where possible, the post should link to the original source and/or provide a Document Object Identifier (DOI) or other universal reference number;
8. The post should contain original work by the post author — while some quoting of others is acceptable, the majority of the post should be the author's own work;
9. Users and readers may report potential abuse of the icons and aggregation system by flagging the post on RB site. Reported abuses may be brought to the attention of readers and discussed publicly online.

There are previous studies about Research Blogging, focusing in its characterization as areas covered, journal titles cited, bloggers' gender and anonymity and other aspects [30], [31]. Our study expands to a closer look to RB, in order to get insights into its contribution to the changes which we verify in scientific communication.

## Methods

### Data collection and treatment

We conducted an exploratory study, with a quantitative approach to guide the search into posts by the Research Blogging Website. The search was performed in January 2012 and included the entire period available in RB since its inception, considering the posts published between November 1, 2007 and December 31, 2011. We chose to analyze only posts actively discussing peer-reviewed articles published in scientific journals, and excluded posts that merely listed references with no discussion. Citations in posted entries with references to books, conference proceedings, guidelines and other online or offline sources were disregarded. We also disregarded those without an active online address and no longer available – only six blogs with a total of 12 posts.

Data were extracted, we hand-searched reference lists from retrieved posts to verify inconsistencies, and then the treated data were summarized in order to generate quantitative descriptions of the following:

*number of blogs
*categories by RB topic
*distribution among the seven languages adopted by RB
*number of posts
*citation distribution (number of articles cited by post, journal titles, in restricted journals and in OA journals)
*reach by number of views.

In addition to generating automatic references on RB by searching for the DOI from scholarly papers, bloggers can create references manually when DOI is unknown, and thus they do not follow a single standard to refer to the journals, *e.g.* the Proceedings of the National Academy of Sciences of the United States of America appears in full, abbreviated by Nat. Acad. Sci. USA, and by PNAS. This was the case of some journals in the extracted sample, and this sort of lapse in standardization is a common problem in data mining for informetric research [32], thus any sample obtained automatically must be checked for find inconsistencies and be previously treated to a valid analysis. Here we confirm the titles by the consultation to Ulrich's Periodicals Directory Online [http://ulrichsweb.serialssolutions.com].

Two of the original topics present in RB, Health and Medicine, were joined into a single topic, Health Sciences, and their data values were added to facilitate the analysis under a single category. For counts of views, we consider unique views for each post, and a view for each article cited in this post; *i.e.* two articles in one post were considered to be viewed two times, while one view was assigned to the post. For all other analysis, we consider simple counts. The access status of periodicals in search for Open Access journals was accessed by consultation to the Directory of Open Access Journals (DOAJ) [http://www.doaj.org, last accessed in January 2012].

### Statistical analysis and comparison among metrics

We counted the blog citations and post visualizations for each scientific journal cited in the RB database. We obtained 7 scientometric measurements for the journals available at Journal Citation Reports (JCR) from Thomson Reuters, namely: Journal Impact Factor, Total Articles, Total Citations, Half-Life, Immediacy Index, Eigenfactor Score and Article Impact. We evaluated the correlations among RB count variables and JCR metrics through the Spearman's ρ statistic. The significance of the correlations was accessed through a permutation test (9,999 permutations) and were evaluated at the level of α = 0.05. Additionally, to investigate if Open Access policy would influence citations (*i.e.* OA articles were more cited than expected), we compared the proportion of OA blog citations to the proportion of OA articles in the sample through a binomial test. These analyses were performed in the R programing environment v. 2.14.2 [33].

## Results

Our results below were extracted from the raw data which are available in Supporting Information [Spreadsheet S1], in accordance with the scenario for science 2.0, with data spreading and sharing [34].

### Totals by Blogs and Posts by RB topics and Journal Titles by Area

**Figure 1:**
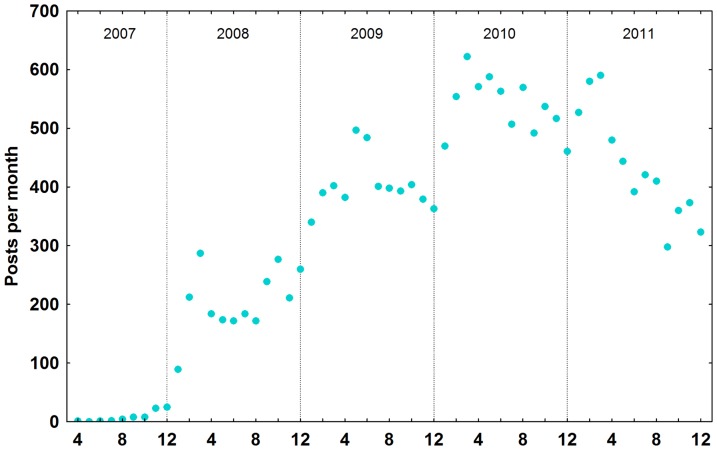
Research Blogging Posts over time. RB posts indexed since its creation.

**Figure 2:**
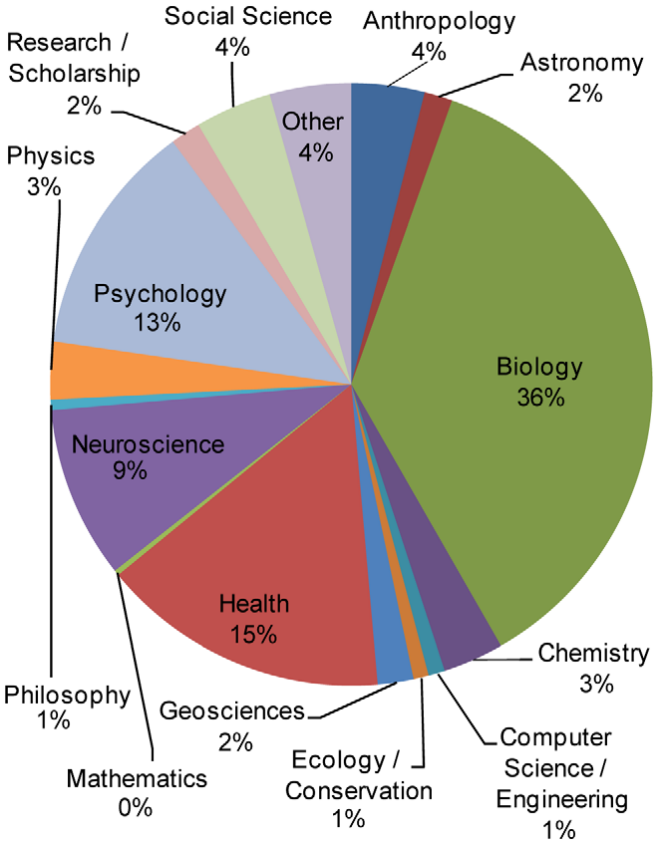
Post distribution by Research Blogging topic category. Posts classified by self-assigned categories available within RB site.

During the period analyzed, the Research Blogging website collected, registered, indexed and shared 26,969 posts by 1,236 blogs considering all entries in total [Fig. 1]. The RB topic with the most posts was Biology, with 9,787 posts (36%), followed by Health Sciences (here combined with Health and Medicine), with 4,177 posts (15%). Psychology had 3,401 posts (13%), Neuroscience had 2,495 (9%), Social Science 1,108 (4%), Anthropology 1,058 (4%), Chemistry 879 (3%), Physics 835 (3%), Geosciences 518 (2%), Research/Scholarship 438 (2%), Astronomy 407 (2%), Computer Science/Engineering 239 (1%), Ecology/Conservation 221 (1%), Philosophy 152 (1%) and finally Mathematics with 77 posts. The Other RB topic category had 1,177 posts (4%) [Fig. 2].

### Language

**Figure 3:**
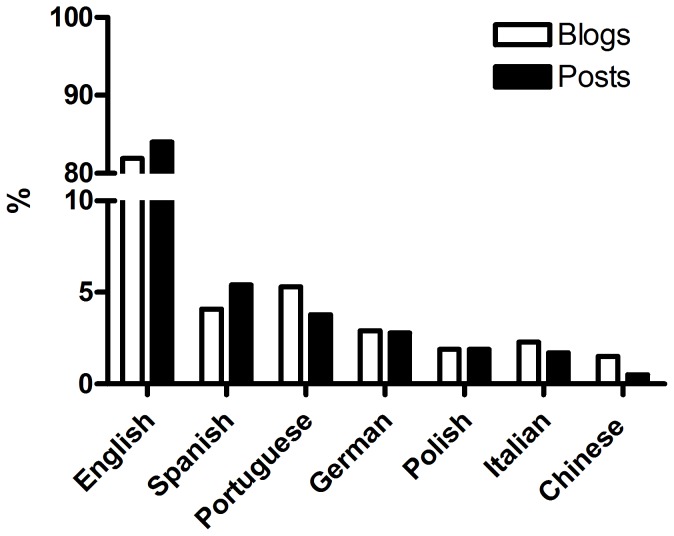
Research Blogging post distribution by language. English is supported since RB inception in 2007. The other languages were added gradually (German, August 2008; Spanish, May 2009; Portuguese, June 2009; Chinese, August 2009; Polish, April 2010; Italian, December 2010).

The most common language was English with 1,008 blogs and 22,660 posts, followed by Portuguese, with 65 blogs and 1,013 posted entries. Spanish had 52 blogs with 1,456 posts, German had 36 blogs and 742 posts, Italian had 32 blogs with 449 posts, Polish had 24 blogs and 512 posts, and Chinese had 19 blogs with 137 posted entries [Fig. 3 and Table 1].

**Table 1:** Research Blogging post topic by language.

Topic/Language	English	Chinese	German	Italian	Polish	Portuguese	Spanish	Total
Anthropology	923	0	16	0	86	1	32	1058
Astronomy	306	0	90	8	0	3	0	407
Biology	8222	11	385	141	64	270	694	9787
Chemistry	518	37	47	25	98	154	0	879
Computer Science/Engineering	208	24	3	2	0	2	0	239
Ecology/Conservation	188	0	0	10	0	7	16	221
Geosciences	441	0	6	0	56	0	15	518
Health Sciences	3790	0	4	15	37	252	79	4177
Mathematics	25	0	19	33	0	0	0	77
Neuroscience	1856	2	0	3	11	132	491	2495
Philosophy	61	0	14	1	0	9	67	152
Physics	517	16	73	174	3	15	37	835
Psychology	3133	36	11	28	49	127	17	3401
Research/Scholarship	425	2	9	0	0	2	0	438
Social Science	1082	9	10	1	3	3	0	1108
Other	965	0	55	8	105	36	8	1177
Total	22660	137	742	449	512	1013	1456	26969

Posts classified by self-assigned categories available within RB site.

### Citations

**Figure 4:**
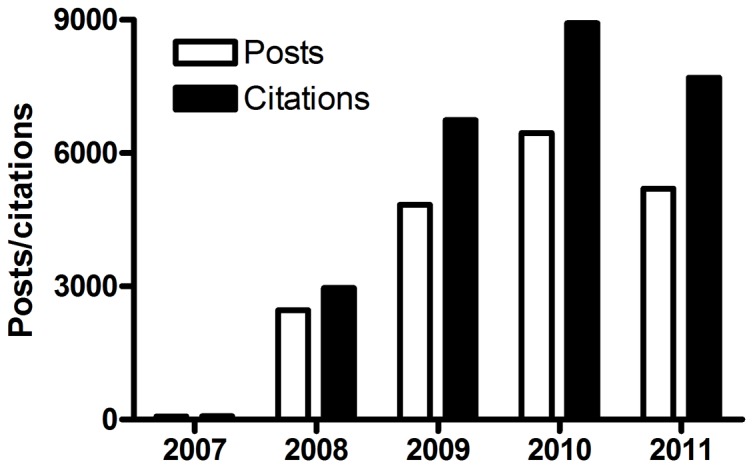
Research Blogging posts and citations. Only posts citing peer-reviewed research from periodicals were considered.

**Figure 5:**
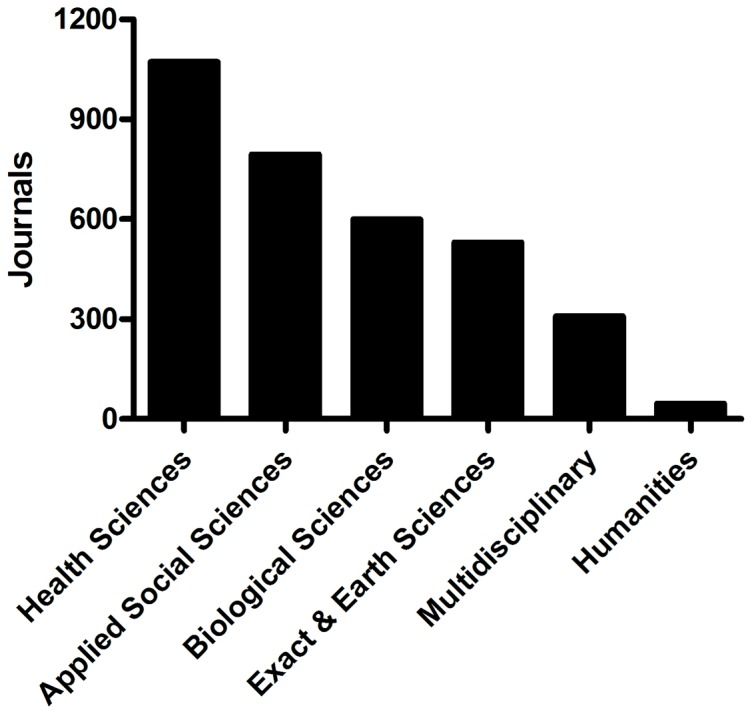
Journal titles by subject areas.

Within the analyzed period 19,000 RB posts cited and linked 26,154 scientific papers published in 3,350 different journals [Fig. 4]. The most-covered subject area by journal titles was the Health Sciences, with 1,071 titles, followed by Applied Social Sciences with 796 titles. Biological Sciences had 599 journal titles, Exact & Earth Sciences, 530 titles while the Multidisciplinary area had 308 titles and the Humanities 46 journal titles [Fig. 5].The journals cited 1,000 times or more were Science (1,829 times), Nature (1,803), Proceedings of the National Academy of Sciences USA – PNAS (1,372) and PLoS ONE (1,156): all general purpose periodicals [Table 2]. This result is similar to the sequence found by Shema, Bar-Ilan & Thelwall [30] in a minor sample of RB posts, putting these four first journal titles in a “Golden Circle” on the Research Blogging website. The citation trend does not follow a close relation to Impact Factors (IF), and all three groups of most cited journals have some periodicals with high IFs and some with IFs of less than 10.

**Table 2:** Most cited Journals at Research Blogging posts.

Group	Citations	Journal Title	IF	Times Cited
First	1,000 times or more	Science	31.364	1,829
		Nature	36.101	1,803
		PNAS	9.771	1,372
		PLoS ONE **OA**	4.411	1,156
Second	201 to 350 times	Proceedings of the Royal Society B: Biological Sciences	5.064	342
		Psychological Science **OA**	4.699	284
		New England Journal of Medicine	53.484	257
		Current Biology	10.025	249
		BMJ	13.660	246
		PLoS Biology **OA**	12.469	242
Third	101 to 200 times	Journal of Personality and Social Psychology	5.205	195
		JAMA	30	154
		Journal of Neuroscience	7.271	154
		Physical Review Letters	7.621	154
		Cell	32.401	151
		The Lancet	33.633	151
		Nature Neuroscience	14.191	137
		Biology Letters	3.651	129
		Pediatrics **OA**	5.391	125
		Animal Behaviour	3.101	115
		Astronomical Journal	4.555	112
		American Naturalist	4.736	111
		Evolution	5.659	111
		Journal of Evolutionary Biology	3.656	105
		PLoS Medicine **OA**	15.617	105
		Journal of Experimental Social Psychology	2.202	102
		Journal of the American Chemical Society	9.023	101
		PLoS Pathogens **OA**	9.079	101
Fourth	51 to 100 times	36 titles	—	—
Fifth	20 to 50 times	134 titles	—	—
Sixth	5 to 19 times	581 titles	—	—
Seventh	2 to 4 times	1,059 titles	—	—
Eighth	One time	1,512 titles	—	—

Journals and Impact Factors (IF) are grouped according to approximate number of citations. Open Access journals are marked with OA.

The second most-cited group of journals spanned from 201 to 350 citations and include six journal titles; the third most-cited group was those with between 101 and 200 citations — 18 titles. There were 36 journals with 51 to 100 citations, 134 journals with 20 to 50 citations, 581 journals with 5 to 19 citations, 1,059 journals with 2 to 4 citations, and 1,512 journals with one citation.

**Figure 6:**
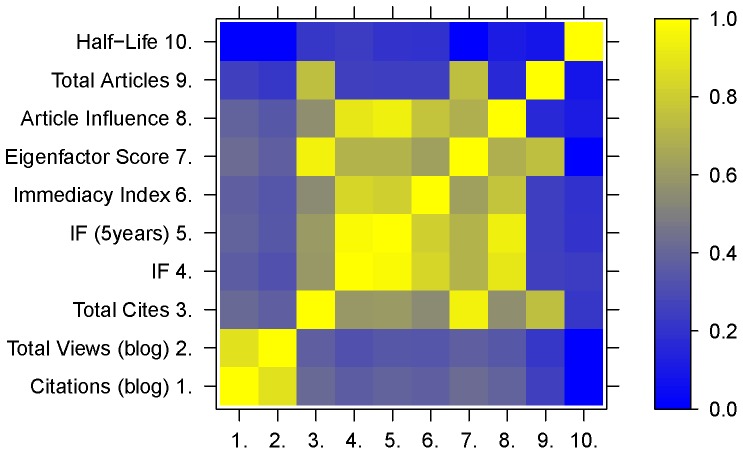
Correlation matrix between RB counts and JCR metrics, depicting the magnitude of correlation between variables. All non-significant correlations were set to zero.

From the 3,350 journals listed in the RB database, 1,822 had scientometric information available at JCR. The correlation matrix shows a moderately modular structure [Fig. 6]. The lowest correlations were associated with Article Half-Life, showing a mean correlation of 0.18 with other metrics and non-significant correlations with both RB count variables. The Total Number of Articles also seems to have generally low correlations with other metrics, with values ranging from 0,16 to 0,26, except for Total Citation and Eigenfactor Score (0.74 and 0.75, respectively). Apart from those variables, all JCR metrics shows correlations among themselves that ranges from 0.54 to 0.97 (0.94, excluding 5year based IF), with an average of 0.58, even if we exclude the 5year based IF. In contrast, RB counts have correlations with the JCR metrics (except Total Articles and Half-Life) that ranges from 0,32 to 0,42, with a mean correlation of 0.37. RB counts showed an average correlation of 0.27 with Total Articles. The correlation between RB citations and RB counts was 0.88.

### Open Access Journals

The results showed that 11.7% of the citations (3,054 of 26,154) came from Open Access journals, a value four times larger than that observed in Wikipedia citations - 2.8% [16]. These citations come from the 7.2% OA journals present in our sample (241 of 3,350). The differences between the proportion of OA citations and OA articles available were significant under a binomial test (p = 2.079e−144). Unlike in Wikipedia citations, six of the most cited journals were OA [Table 2]: PLoS ONE in First group (with more than 1,000 citations), Psychological Science and PLoS Biology in Second group (with between 201 and 350 citations) and PLoS Medicine, Pediatrics and PLoS Pathogens in Third group (with 101 to 200 citations). Also, when visits were considered, three of the 10 most visited article links were of OA journals: PLoS One, Psychological Science and PLoS Biology.

### Reach

**Figure 7:**
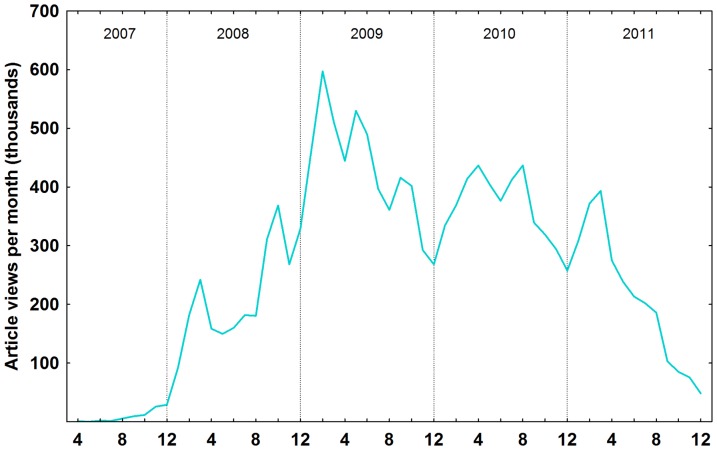
Total article views per year. Article views (in thousands) are represented according to citing posts at Research Blogging. Most recent articles have less time to accumulate views.

**Figure 8:**
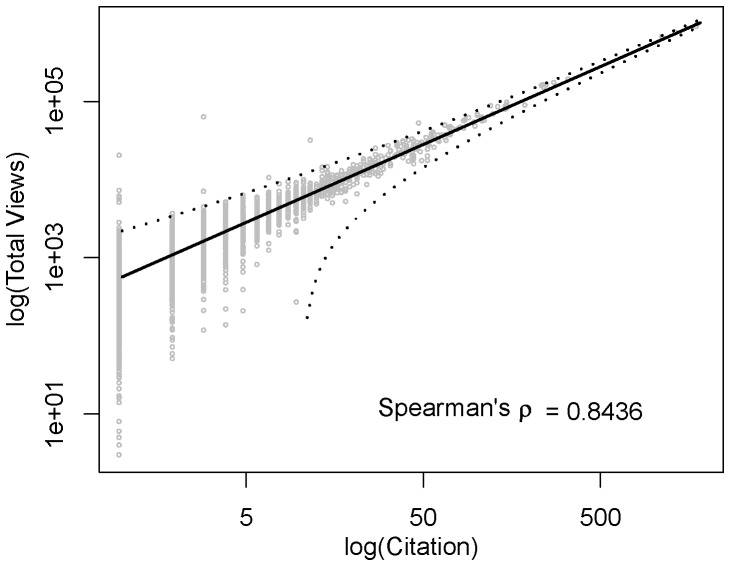
Relationship between RB Total Views and Citations. The trend-line was estimated through exponential fitting between the count data through non-linear squares and the correlation was estimated through Spearman rank-based statistic ρ.

As explained in Methods, for view count we considered unique views for each post, and a view for each article cited in this post, *i.e.* to two articles in one post were two separate views, one for each article [Fig. 7], and only one view for the post. As expected, results showed that more cited journals obtained higher numbers of overall views, but this is also true for some less cited journals, which obtained high number of views too [Fig. 8]. The opposite trend was found to individual article from journals often cited that in some cases did not obtain a high number of views. When we analyzed the views for unique articles - not journals - some surprising views were seen: the most-viewed article was from the Journal of Applied Animal Welfare Science, which has an IF of only 0.71. It received 62,217 views, well ahead of second place, an article in Proceedings of the Royal Society B: Biological Sciences (IF 5.064), which had 15,265 views.

## Discussion

### Blogs and Posts by RB topics

Both the registered blog totals and total number of posts are dominated by Biology (36% of posts). Health Sciences appears in a distant second position (15%), an interesting result since the area of Health Sciences dominates scientific communication, in a number of traditional publications [35], also verified here when we analyze the cites by the journal titles [Fig. 5]. Other categories have minor representation, with 13% (Psychology) and 9% (Neuroscience). Still others form a long tail of the site, with the remaining eleven categories taking less than 4 percent of the total number of posts on RB [Fig. 2]. It's possible that the topic distribution is due to the early dominance of Biology. Perhaps other disciplines saw RB as primarily a Biology/Health site and opted out.

### Frequency of posts per year

The frequency of posts grew vigorously from the establishment of Research Blogging in November 2007, with the number of posts in 2009 doubling over 2008 [Fig. 1]. After a peak in 2010, in 2011 the number of posts declined to levels similar 2009. This increase in 2010 may be related to Research Blogging Awards 2010, since nominations started early February and winners were announced early March, 2010. Following this period, despite the addition of new blogs and languages, the number of posts and views have returned to values equal to or smaller than 2009. We consider the equivalency in posts from 2011 and 2009 an actual decrease in blog posts, since this number results from more blogs and languages that adopted RB during these two years. During the second half of 2011, the automatic aggregation tool of RB was not functional, which may have led to this decrease. This difference could be in part explained by a shift in science divulgation in recent years from blogs to other online platforms, such as social networks (*e.g.* Twitter and Facebook). These tools have different purposes and functionalities, and mainly in the case of science writing would be more a good complement for spreading scientific blog posts [36] and to collect and share stories and resources [37],[38] rather than construction and discussion as observed in blogs, indicating that these new tools are more related to social networks acting in spread and sharing information, linking to contents (including to the blogs), whereas blogs would be considered as information repositories. With faster ways of propagation and discussion of topics in these new tools, the decrease in post numbers may represent shorter comments on articles left out of blogs and posted in social media instead, and that posts are less frequent but used to cover research more thoroughly.

### Languages and RB Topics covered

The dominant language on RB is English, with 1,008 blogs posting 22,660 entries, followed by Portuguese (65 blogs and 1,013 posts), Spanish (52 blogs and 1,456 posts), German (36 blogs and 742 posts), Italian (32 blogs and 449 posts), Polish (24 blogs and 512 posts), and Chinese (19 blogs and 137 posts) [Fig. 3, Table 1].

English has been supported for the longest period at RB, having been a part of the system since its inception in 2007. The other languages were added gradually (German, August 2008; Spanish, May 2009; Portuguese, June 2009; Chinese, August 2009; Polish, April 2010; Italian, December 2010), and there is some correlation between when a language was added and the number of posts in that language. However, perhaps because more science publishing and blogging occurs in English, or because the RB interface is in English, English continues to substantially outpace the other languages.

There are also some interesting regional patterns. The general dominance of Biology is not the same in all languages. In Italian, there are more posts in Physics than Biology (174∶141), in Chinese there are more posts about Chemistry (37), Psychology (36) and Computer Science/Engineering (24) than Biology (11). Polish demonstrated an equilibrated distribution of topics [Table 1]. These regional peculiarities show an interesting avenue for future comparisons in scientific communication among different cultures.

### Citations

Our correlation analysis shows that there is general decoupling of blog metrics and other classical scientific metrics (Fig. 6). This is exemplified by the fact that, generally, the correlation between RB counts and JCR metrics are lower than those observed among the majority of JCR metrics, with the exception of Article Half Life and Total Articles. This suggests that the main factors influencing journal citation in the blogosphere are not the same that determine journal merit, as evaluated through JCR metrics, even though academic merit have a substantial influence on blog citation, as reveled by the presence of significant correlations among almost all JCR metrics and RB counts.

The presence of this imperfect association between classical metrics and blog citation can be exemplified by the fact that high IFs are present in most-cited journals but are not a prerequisite or predictive of journal citations in posts [Table 2]. So, rather more than being more frequently cited due to high relevance due to IF, the “Golden Circle” may also be favored because it consists of multidisciplinary journals, while those with fewer citations are specialized journals, with a more restricted audience. We consider the wide variety of journals that were discussed to be a positive feature of RB, although almost half of the titles was only referenced once in the study time period.

These findings in general draw attention to the importance of new article level metrics and other scientometrics tools for measuring the relevance of papers outside traditional publications [19]–[21]. Also, as articles cited in Wikipedia tend to be more relevant than equivalents, an indicative that the choice of Wiki citations favors relevant research [16], it may be interesting to follow if citations in blog posts are predictive of future article relevance.

Another interesting finding was that increasingly blogs cite more articles in the same post. One post had 29 citations, 18 of which refer to articles that are part of a series derived from a project proposal to the National Science Foundation (NSF) [39]. This difference supports the argument that blogs promote a deeper understanding of the subjects they cover and the hypothesis that bloggers are writing less frequently, but dealing with more relevant information. In fact, while the number of posts and citations fell in absolute terms in 2011 [Fig. 2, 3], the number of citations per post did not. This number has increased from 1.38 in 2010 to 1.48 in 2011, which may indicate that bloggers are beginning to add more content to each post. Also, blogs tend to cite more types of sources than just peer-reviewed articles, leading to questions about online metrics: Are mentions of published scientific research at blogs or Wikipedia as valid as citations? Should we reconsider what we commonly understand by citation: an article talking about another article? These are important questions, since the process of scientific communication is historically based on procedures which don't necessarily have analogs in a digital and 2.0 context, where we are looking for new, valid metrics for assessing the reach and impact of science and research [40].

### Open Access journals

There is a large, ongoing effort to promote and disseminate Open Access scientific journals, motivated by the idea that scientific information must flow freely to generate more knowledge [41]. Our findings show that the number of OA journals cited by RB posts is much larger than observed for Wikipedia citations [16], suggesting that blog authors have favored OA content, and blog readers have proportionally more access to the original article discussed at the posts. Recently, there has been an increasing concern in publication policy and public access to research results [42], [43], and academic bloggers are especially engaged in these matters [44], which may reflect in OA trends. The large presence of paid content journals indicates that bloggers still maintain some of the characteristics of traditional scientific discourse as a preference for high-impact and multidisciplinary journals, following findings in others studies about RB [12], [30]. On the other hand, we suggest that they perform an important social function by exposing and explaining scientific content that is inaccessible to the general public due to the constraints of paid access scientific journals in a transition context permeated by the effort to the greater access to scientific knowledge.

### Reach

The results corroborate the methodologies of Article-Level Metrics that consider the individual article to determine its value and reach, in contrast to journal-level measures of research quality that have traditionally been made available until now [19], as an alternative form to verify the quality, importance, and relevance to scientific literature, more immediately than the IF allows. One of the criteria of article-level metrics - the number of views to the article - allows verify the article relevance soon after the publication unlike journal-level measures based in IF.

In addition, as P. Janiszewski points out, citation on blogs may improve the reach of research:

Put another way, the same research which I published in a prestigious medical journal and made basically no impact, was then viewed by over 12,000 sets of eyes because I decided to discuss it online. And it doesn't end there [45].

The systematic indexing and citation registering adopted by RB is an efficient filter for published research and its dissemination, allowing article views and access statistics agree with blog coverage metric [20].

### Future directions

Extracting data from RB posts is a challenge, mainly due to the heterogeneous pattern of journals entries by the bloggers, as previously explained. Also regarding RB further improvements, it will be useful to allow its data to be mined by integrating features like its Twitter app with tools like CrowdoMeter [46],[47], improving the categorization of the citations in RB posts, and integrating other tools to promote a joint effort with the scientific community. Additionally, it would be informative to deeper evaluate the regional patterns observed between languages, allowing comparisons in scientific communication among different cultures.

The emergence and rise of more recent online technologies and services based in social media tools such as Twitter may mean that blogs, one of the oldest digital platforms, are losing ground in numbers. We believe that blogging is still an important way to give visibility to science in a more complete and detailed format. It can offer an alternative view of science, one that is more transparent, comprehensive, and comprehensible, while increasing interest, usage and reach of scientific publications; it continues to hold an important place among other new technologies. Platforms like RB not only spread but also record and index published research, as well as having an important social function by bringing restricted publications of science to the general public.

Also, it points to a new path of scientific information spreading. The previous (and somehow still ongoing) path was: 1) scientific data published in traditional scientific journals; 2) press releases; 3) scientific data divulged (not always accurate) in the mass media. An important new ongoing path is: 1) scientific data published in traditional scientific journals and also in open access scientific journals; 2) peer-reviewed posts published in science blogs, which provides updated and accurate scientific information in more accessible language to a non-scientific public. Considering this, it would be a relevant challenge to develop and/or improve new metrics related to tools like RB in order to better evaluate its effective contribution to scientific information reach.

In this sense, our correlation analyses suggest that RB citations and views indeed evaluate different aspects of scientific production. The fact that the correlations between RB counts and JCR metrics is lower then the correlations among JCR variables (with the exception of Total Articles and Article Half-Life) suggests that the overall factors influencing the traditional metrics are not the main factors in defining blogging citations and views. If the pattern found here for JCR metrics are consistent with large-scale studies of correlation between different metrics [48], than this could be an indicative that RB-based metrics are evaluating a different feature of journal quality, merit or impact. Even if RB counts are connected to Usage metrics (*e.g*. Closeness Centrality, Degree Centrality, Journal Use Probability), the mean correlation between those and Citation metrics is very high (according to Bollen et al [48], it ranges 0.68 to 0.73, with the exception of Usage Impact Factor, with a value of 0.27), strongly suggesting that RB counts are evaluating a different aspect of research quality. Specific investigations of the relationship between Usage metrics and RB counts are warrant in order to evaluate the true relation of these metrics. Overall, RB metrics correlations are consistent with findings for other altmetrics [49], indicating that they should be viewed as such.

Even though RB counts would not be available to all journals (not all journals are cited in blogs), they nevertheless state something about the social impact of those that were cited, and could be of use to journal editors that wish to develop policies to increase their journal outreach. Large publishers (such as Nature group) are already doing this through the establishment of a blogosphere linked to their publications. RB is different in this sense because it is not directly connected to any scientific publishing group and could be seen as a relatively independent source of scientometric information, and a more reliable base for policy-making.

## Supporting Information

Spreadsheet S1 **Research Blogging Reports raw data.** Excel spreadsheet with Research Blogging data from November 1, 2007 until December 31, 2011. **Sheet S1-A**: RB Blog Report with blog name, blog URL, status, Research Blogging topic, number of posts and blog language. **Sheet S1-B**: RB Citations Report with publication date, post title, number of views, blog name, DOI, journal title and Research Blogging topic.
(XLSX)
