# Correction: Research Blogging: Indexing and Registering the Change in Science 2.0

**DOI:** 10.1371/journal.pone.0124184

**Published:** 2015-04-07

**Authors:** The PLOS ONE Staff

There are errors in the Funding section. The correct funding information is as follows: FAM and AI are supported by FAPESP (Fundação de Amparo a Pesquisa do Estado de São Paulo), grant numbers 2011/21674-4 and 2008/58559-5, respectively.
